# Congressman John E. Fogarty: A Champion for Global Health

**DOI:** 10.4269/ajtmh.17-0612

**Published:** 2017-09-07

**Authors:** Mary Fogarty McAndrew

It was just a few short years ago that we celebrated the Centennial of my Dad’s birth. That was in 2013 and it is now a little over 50 years since my Dad died at his desk just before being sworn in as a Member of Congress for his 14th term. A lot has happened since then.

**Figure:**
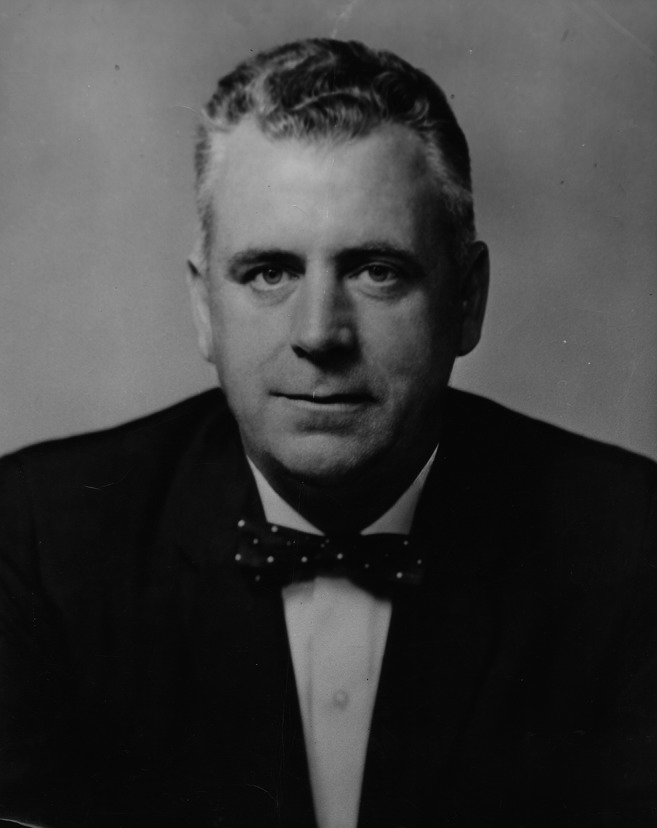
**Congressman John E. Fogarty (1961)**

**Figure:**
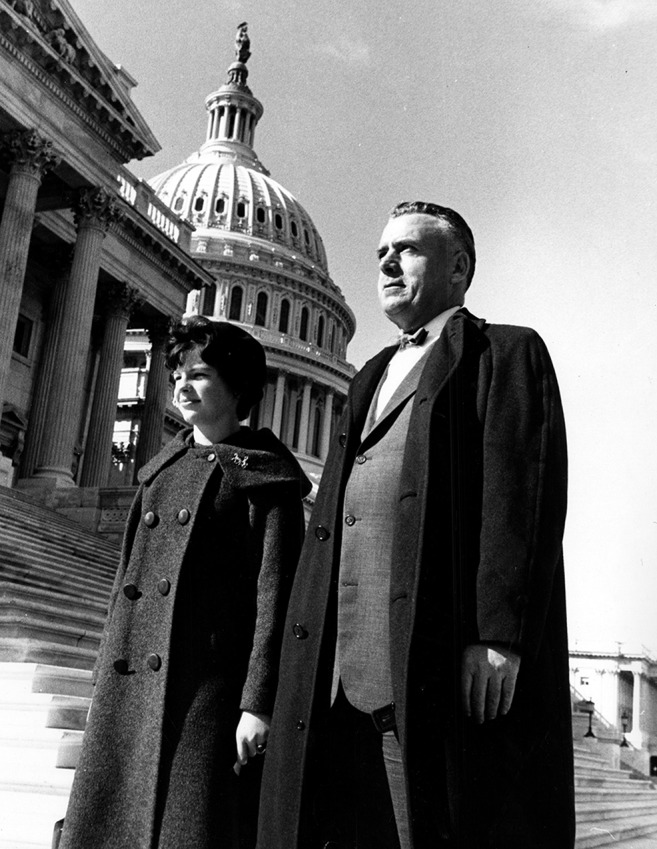
**Fogarty on the steps of the U.S. Capitol with his daughter, Mary Fogarty McAndrew (March 1962).**

**Figure:**
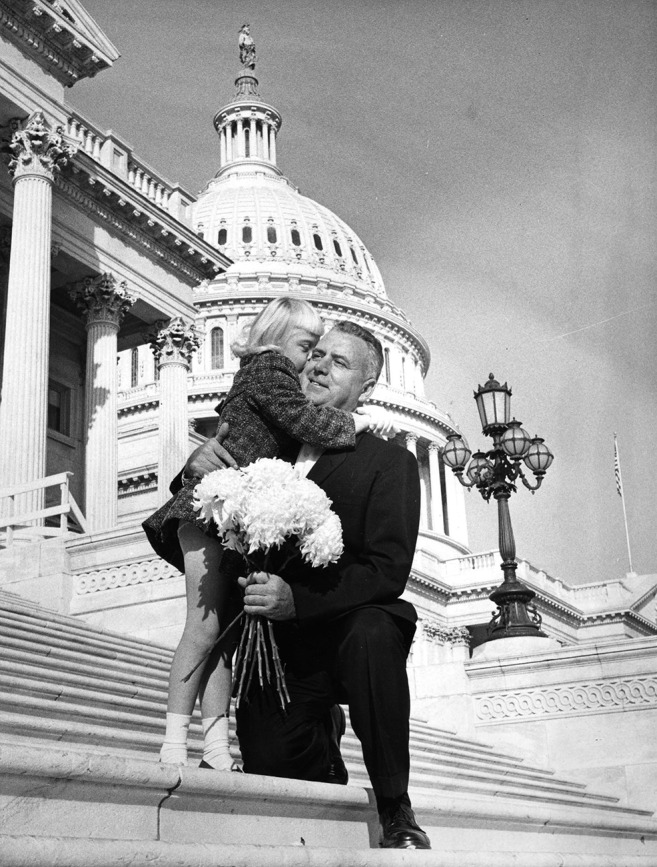
**Fogarty on the U.S. Capitol steps with a young girl (1963). In addition to his work on global health, he was also known as an advocate for children with intellectual and developmental disabilities.**

My father was born on March 23, 1913, the third of six children. He grew up in a town called Harmony in the northern part of Rhode Island. He had planned to enroll at the College of the Holy Cross, but the Great Depression changed that. Rather, he joined his older brother and father in bricklaying, a profession that prepared him very well for the arduous work that awaited him in the U.S. House of Representatives. He went from being the president of the local Bricklayer’s Union, No. 1 in 1939 to being elected to represent the Second District of Rhode Island in 1940.

In 1947 he was appointed to the House Appropriations Committee to serve on the Subcommittee on Labor, Health, Education and Welfare, where he would become the Committee’s youngest chairman in 1949. R.I. State Historian Laureate Patrick Conley said:

“… just like Paul of Tarsus was suddenly transformed on the road to Damascus, John Fogarty was transformed on the road to Bethesda…John believed that one ounce of prevention was worth a pound of cure; in other words 1 billion for medical research might save 16 billion in medical care.”

And, my Dad never looked back. He truly believed that “no man is an island” and that collaborating with colleagues at home and abroad brought the best minds together to attack the pestilence, war, famine, and death that still plague millions of people around the world today. He said, “As long as people are sick, something has to be done to make them better. The government has to give most of the help because there’s no one else to give it.” He was called a “health zealot” by some and took that as a compliment. As a result of his untiring dedication to better health he would become known as Mr. Public Health:

“As we limit the span of uncertainty in the cause of death and illness and extend and enrich the span of life, we act in the highest ideal of government in the service of the governed, and in the best tradition of public, private, and individual enterprise.”

I think it is safe to say that we all know someone who has benefited from the work that my father and his Congressional colleagues did to further medical research and to build the premier national biomedical research facility—the National Institutes of Health (NIH). During his 26 years in Congress he expanded funding for the NIH from $28.5 million in 1949 to $1.1 billion at the time of his death in 1967 at the age of 53. It is very important to note that my father did not do this alone. My Dad had the good fortune to have his dear friend Mel Laird from Wisconsin as his Ranking Minority Member. Together, they shaped public health policy and routinely increased funding for medical research, often beyond what their Presidents and fellow lawmakers thought was sufficient. According to my Dad, “There are no politics in this committee because these departments affect every human being in our country.” And on the Senate side they had Lister Hill of Alabama, who along with my Dad sponsored the Hill-Fogarty “Health for Peace” bill, opening up further opportunities for the support of research and training on an international basis.

My Dad argued that increasing federal support for medical research reduced human suffering and was an “economical investment in life…Research is the only means we have for reducing the growing federal burden of medical care costs.” In the midst of the Korean War and the intensifying Cold War he knew this research would benefit our country’s defense by increasing the number of healthy people who could serve in our military. He also stressed the importance of being able to combat bacteriological warfare and address treatment in the field from injuries caused by new weaponry, all of which would contribute directly to our national security. Increased medical research had similar benefits for our economy: healthier workers and reduced disabilities. These outcomes would in turn contribute to peace in the world. We would also benefit economically if people of other nations were healthier because U.S. trade would increase and we would not have to send so much money in aid to other countries. He saw early on the need to work with all nations to curb the transmission of disease, a point that was painfully brought home by the Ebola outbreaks of recent memory. He truly knew the importance of global health, stating:

“I have always recognized…that just as disease knows no national boundaries so also the benefits of medical research and indeed research itself can know no boundaries. Time and time again, it has been demonstrated that the goal of better health has the capacity to demolish geographic and political boundaries and to enter the hearts and minds of men, women, and children in the four corners of the earth.”

It was difficult to believe that in 1962 my Dad was talking about a world that had shriveled in size, with the most distant places only hours apart. My father, indeed, had a prescient vision of how important global health was and would be. As far as global health is concerned, the genie is out of the bottle; the world is smaller today and continues to shrink. Global health has never been more vital than it is today and its importance will only increase. The NIH, the preeminent medical research facility in the world, must have a substantial organization dedicated to global health. This is the Fogarty International Center, which supports basic, clinical, and applied research and training for U.S. and foreign investigators working in the developing world. The Center serves as a bridge between the NIH and the greater global health community. Since its establishment in 1968 through legislation introduced by Melvin Laird right after my Dad’s death, nearly 6,000 scientists worldwide have received significant research training through Fogarty programs that benefit people here at home and around the world.

My Dad was a delegate to World Health Organization meetings for many years and firmly believed that major investments in health around the world could and would lead to peace. I agree, as he stated:

“I think that this matter of expanding research is one – perhaps the one – truly global effort in which all nations can and will join as real partners.”

I recently had the good fortune to meet with Representative Tom Cole (R, OK), the Chairman of my father’s subcommittee, and found him to have the same philosophy and concern for global health as my father. Chairman Cole would rather “fight Ebola in West Africa than in West Dallas” – so would my Dad.

